# The Impact of Rhinosinusitis and Its Treatment on Sleep-Disordered Breathing: A Systematic Review

**DOI:** 10.3390/medicina62071392

**Published:** 2026-07-18

**Authors:** Taif Mansour Almaqboul, Amal Kharallah Almutairi, Lujain Barkheel Albarkheel, Atheer Mohammed Alshammakhi, Rehaf A. Areeshi, Abdullah Ahmed Alarfaj, Khalid AlYahya

**Affiliations:** 1 College of Medicine, Batterjee Medical College, Jeddah 21442, Saudi Arabia; 2 College of Medicine, Majmaah University, Al-Majmaah 11952, Saudi Arabia; amal2almutairi.1@gmail.com (A.K.A.); lujain.br3@gmail.com (L.B.A.); 3 College of Medicine, Jazan University, Jazan 45142, Saudi Arabia; atheeralshammakhi@gmail.com (A.M.A.); rehaf.areeshi@gmail.com (R.A.A.); 4 Otorhinolaryngology Unit, Surgery Department, King Faisal University, Al-Ahsa 31982, Saudi Arabia; aalarfij@kfu.edu.sa (A.A.A.); kalyahya@kfu.edu.sa (K.A.)

**Keywords:** rhinosinusitis, sleep-disordered breathing, apnea-hypopnea index, nasal obstruction, endoscopic scores

## Abstract

*Background and Objectives*: Rhinosinusitis contributes to the development of sleep-disordered breathing (SDB) by causing nasal obstruction and airway inflammation; however, the extent to which treatment improves these conditions remains unclear. To address this, this study systematically evaluated the impact of rhinosinusitis-directed therapy on nasal symptoms, endoscopic findings, and sleep-related respiratory parameters. *Materials and Methods*: Methodologically, this systematic review followed the Preferred Reporting Items for Systematic Reviews and Meta-Analyses (PRISMA) 2020 guidelines. A comprehensive search was conducted across the PubMed, Medline, Web of Science, Science Direct, and Google scholar databases. Specifically, studies reporting pre- and post-treatment outcomes in patients with rhinosinusitis were included. Due to substantial clinical and methodological heterogeneity among the included studies, a quantitative meta-analysis was not performed; instead, findings were synthesized narratively. *Results*: Ultimately, fifteen studies met the inclusion criteria and demonstrated consistent improvements in sleep-related outcomes following the treatment of rhinosinusitis. Across the surgical and medical interventions, reductions in apnea-hypopnea index (AHI) and improvement in endoscopic findings were frequently reported. For instance, functional endoscopic sinus surgery (FESS) produced notable enhancements in sleep quality, daytime sleepiness, and sinonasal symptom scores in several studies, while biologic therapy and medical management also showed benefit in reducing nasal obstruction, rhinorrhea, and inflammatory markers. *Conclusions*: The available evidence suggests that rhinosinusitis treatment may be associated with improvements in measures of sleep-disordered breathing (SDB) and mucosal inflammation, although subjective nasal symptom outcomes were more variable across studies. Consequently, targeted sinonasal therapy may play an important role in reducing SDB burden, particularly in patients with inflammatory airway compromise.

## 1. Introduction

Sleep-disordered breathing (SDB) encompasses a range of disorders characterized by abnormal respiration during sleep, including primary snoring, upper airway resistance syndrome (UARS), and obstructive sleep apnea (OSA) [1]. These conditions represent a significant public health concern due to their widespread prevalence and serious health consequences. OSA, in particular, affects an estimated 9% to 38% of adults globally and is strongly linked to cardiovascular disease, hypertension, insulin resistance, neurocognitive decline, and a diminished quality of life [2,3].

Chronic or otherwise, rhinosinusitis, with or without nasal polyps, is a common inflammatory condition of the paranasal sinuses and nasal mucosa that can profoundly impair nasal airflow [4]. Chronic mucosal inflammation, mucosal thickening, and structural changes, such as turbinate hypertrophy and sinus ostial obstruction, contribute to increased nasal resistance and mouth breathing; these are pathophysiological mechanisms that predispose the patient to or exacerbate SDB [3,4]. In addition to mechanical obstruction, inflammatory mediators and mucosal edema may alter upper-airway collapsibility and neuromuscular control, thus influencing the occurrence and severity of sleep-related breathing disturbances [5].

Emerging evidence suggests that patients with rhinosinusitis frequently report sleep disturbances—insomnia, excessive daytime sleepiness, and non-restorative sleep, even in the absence of confirmed OSA [6,7]. Medical interventions such as endoscopic sinus surgery (ESS), topical corticosteroids, or biologic therapy, which aim to restore nasal function, have been associated with varying degrees of improvement in sleep quality and SDB parameters [6,7,8,9]. However, the strength and consistency of this evidence remain uncertain because studies differ in design, diagnostic criteria, and outcome assessment.

Given the growing recognition of the bidirectional relationship between sinonasal inflammation and sleep physiology, a systematic synthesis of the existing literature is warranted. This systematic review aimed to synthesize evidence regarding both the association between rhinosinusitis and sleep-disordered breathing (SDB) and the impact of rhinosinusitis-directed treatment on sleep-related outcomes. Specifically, we evaluated whether rhinosinusitis severity, phenotype, and treatment modalities influence sleep-study parameters and patient-reported sleep outcomes.

## 2. Materials and Methods

### 2.1. Search Strategy

This systematic review was conducted in accordance with the Preferred Reporting Items for Systematic Reviews and Meta-Analyses (PRISMA) 2020 [10] guidelines (Supplementary Material) to ensure methodological rigor, transparency, and reproducibility. The protocol was registered in the International Prospective Register of Systematic Reviews (PROSPERO; registration number CRD 420251178797) [11]. A comprehensive literature search was performed on the PubMed, Medline, Web of Science, Science Direct and Google scholar databases from inception through 31 October 2025. The search strategy combined Medical Subject Headings (MeSH) and free-text terms related to rhinosinusitis and sleep-disordered breathing (SDB). The studies were chosen using the web and mobile applications of Rayyan (Rayyan Systems Inc., Cambridge, MA, USA) [12]. The following key concepts were applied:

(“Rhinosinusitis”[Mesh] OR “Chronic Rhinosinusitis” OR CRS OR CRSwNP OR CRSsNP)

AND

(“Sleep Apnea Syndromes”[Mesh] OR “Obstructive Sleep Apnea” OR OSA OR “Sleep-disordered Breathing”)

AND

(“Treatment Outcome” OR “Endoscopic Sinus Surgery” OR FESS OR biologics OR corticosteroids).

No language or study-design filters were applied a priori to maximize search sensitivity. Titles, abstracts, and full-text articles were screened using the Rayyan web and mobile application (Rayyan Systems Inc., Cambridge, MA, USA) to facilitate blinded, duplicate screening and reduce reviewer bias.

### 2.2. Eligibility, Data Extraction, and Management

Two reviewers independently screened all records in duplicate based on pre-specified inclusion and exclusion criteria. Eligible full-text studies were retrieved for detailed evaluation. Data were extracted independently using a standardized, piloted form that captured citation details, study design, patient demographics, sample size, intervention type (medical vs. surgical), sleep-related outcomes, and follow-up duration.

Disagreements between reviewers were resolved through discussion or adjudication by a third reviewer.

### 2.3. Inclusion and Exclusion Criteria

Diagnosis of chronic rhinosinusitis (CRS) in the included studies was generally based on established clinical criteria, most commonly physician-diagnosed CRS supported by nasal endoscopy and/or computed tomography findings, consistent with internationally recognized guidelines. However, minor variability in diagnostic definitions across studies was noted and is acknowledged as a potential source of heterogeneity.

Obstructive sleep apnea (OSA) severity was primarily classified using standard apnea-hypopnea index (AHI) thresholds: mild (5–14.9 events/h), moderate (15–29.9 events/h), and severe (≥30 events/h). Where reported, AHI was measured using in-laboratory polysomnography or validated home sleep testing systems. Differences in sleep assessment modalities across studies were recorded and considered during the interpretation of results.

Studies were eligible for inclusion if they investigated patients of any age diagnosed with rhinosinusitis, encompassing both acute and chronic forms, with or without nasal polyps. Eligible studies evaluated either medical or surgical interventions aimed at treating rhinosinusitis, including but not limited to intranasal corticosteroids, systemic antibiotics, biologic therapies, or functional endoscopic sinus surgery (FESS). Comparisons of interest included pre-treatment versus post-treatment outcomes within the same cohort, as well as comparative analyses between different therapeutic modalities, such as medical versus surgical management.

To be included, studies were required to report at least one sleep-related outcome relevant to sleep-disordered breathing (SDB). Eligible study designs comprised RCTs, cohort studies, case–control studies, cross-sectional studies, and detailed case reports that provided quantifiable or descriptive data linking rhinosinusitis and sleep outcomes. Only human studies published in English and with adequate methodological quality and reporting completeness were considered for inclusion.

Studies were excluded if they focused on sleep disorders unrelated to rhinosinusitis, such as idiopathic obstructive sleep apnea, central sleep apnea, narcolepsy, or other primary sleep pathologies not linked to sinonasal disease. Animal studies, in vitro experiments, and non-human research models were likewise excluded. Systematic reviews, meta-analyses, narrative reviews, editorials, letters to the editor, and conference abstracts without full-text availability were also excluded. Risk-of-bias assessments informed interpretation of the narrative synthesis.

The research question was structured according to the PICO framework.

The population (P) included pediatric and adult patients diagnosed with rhinosinusitis, encompassing acute and chronic forms, with or without nasal polyps.

The interventions (I) comprised medical, surgical, and biologic treatments directed at rhinosinusitis, including intranasal or systemic corticosteroids, antibiotics, biologic agents, functional endoscopic sinus surgery, and other nasal surgical procedures.

Comparators (C) included pre-treatment versus post-treatment assessments within the same cohort, as well as comparisons between different treatment modalities or untreated control groups where available.

The outcomes (O) of interest included sleep-related measures relevant to sleep-disordered breathing, comprising both objective and subjective outcomes. Objective outcomes included physiological and polysomnographic parameters such as the apnea-hypopnea index and other sleep study measures, whereas subjective outcomes included patient-reported measures of sleep quality, daytime sleepiness, and sleep-related quality-of-life scores.

### 2.4. Statistical Analysis

We used Review Manager (RevMan) 5.4 for preliminary data exploration; however, a meta-analysis was ultimately not conducted due to substantial heterogeneity across study aims, outcomes, and methodologies. Risk of bias was assessed according to study design. Randomized controlled trials were evaluated using the Cochrane Risk of Bias 2 (RoB 2) tool, non-randomized intervention studies using ROBINS-I [13], and observational cohort studies using the Newcastle–Ottawa Scale (NOS). Each tool was selected because it was specifically designed for the methodological characteristics of the corresponding study design. Funnel-plot inspection indicated further inconsistency and potential publication bias. Given these limitations, a narrative synthesis was deemed more appropriate than quantitative pooling.

## 3. Results

**Figure 1:**
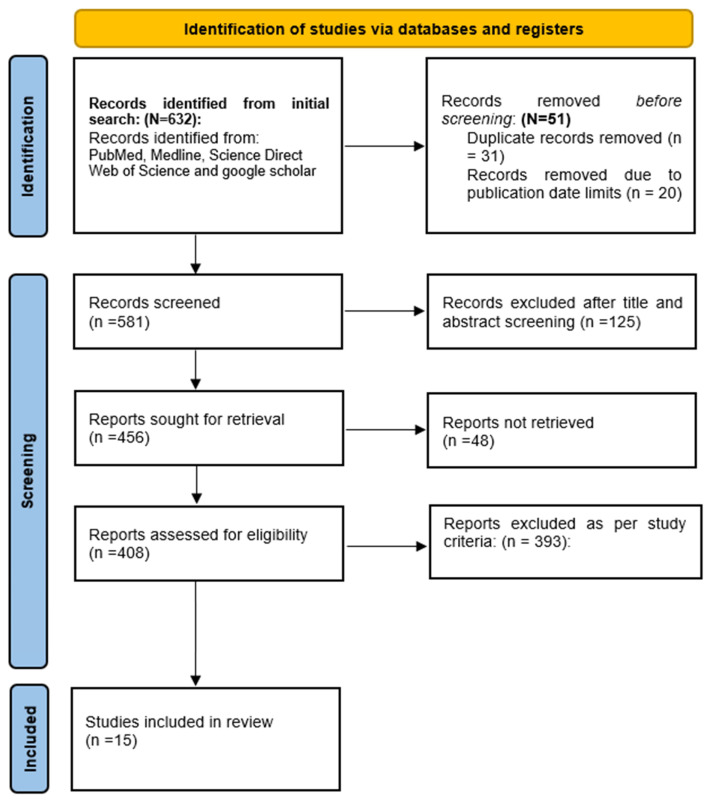
Study selection process according to the PRISMA 2020 guidelines.

The initial search yielded 632 papers from several databases: PubMed (260), Medline (70), Web of Science (30), Google Scholar (215), and Science Direct (57). Following screening of the studies using the aforementioned eligibility criteria, 15 studies were ultimately found to be eligible for inclusion and analysis, as shown in Figure 1.

### 3.1. Study Characteristics

A total of 15 studies met the eligibility criteria and were included in this systematic review. These studies comprised a range of designs, including randomized controlled trials, prospective and retrospective cohort studies, and observational analyses. Sample sizes varied considerably, from small surgical cohorts to large database-driven populations. Interventions included medical therapies, endoscopic sinus surgery, septoplasty, and biologic treatment, while several studies also assessed non-intervention cohorts to explore associations between rhinosinusitis and sleep-disordered breathing. Key study characteristics, interventions, and main findings are summarized in Table 1.

### 3.2. Risk of Bias Assessment

The ROBINS-I assessment demonstrated variability in the risk of bias across the included non-randomized studies. Four studies were rated as having a serious risk of bias, primarily due to concerns related to confounding and incomplete adjustment for baseline differences. The remaining studies were judged to have a moderate risk of bias, indicating some methodological limitations but acceptable control of key bias domains. Notably, none of the studies achieved a low overall risk of bias, highlighting methodological constraints within the available evidence base (see Table 2).

According to the Newcastle–Ottawa Scale, the evaluated studies showed different bias risks. Three studies (Hui et al., Kim et al., Laababsi et al.) were assessed as “Moderate Risk”, and one study, Jiang et al., was rated as “Low Risk”. It was determined that one study, Poachanukoon et al., had a “High Risk” of bias (See Table 3).

### 3.3. Cochrane Risk of Bias Assessment of the Included RCT

**Figure 2:**
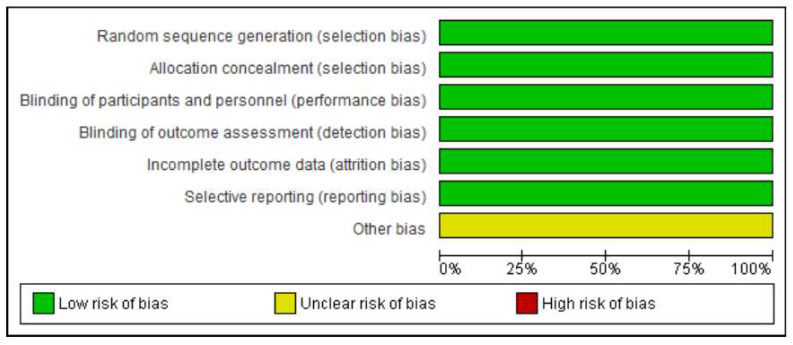
Risk of bias graph summarizing the reviewers’ judgments about each risk of bias domain presented as percentages across all included randomized controlled trials.

**Figure 3:**
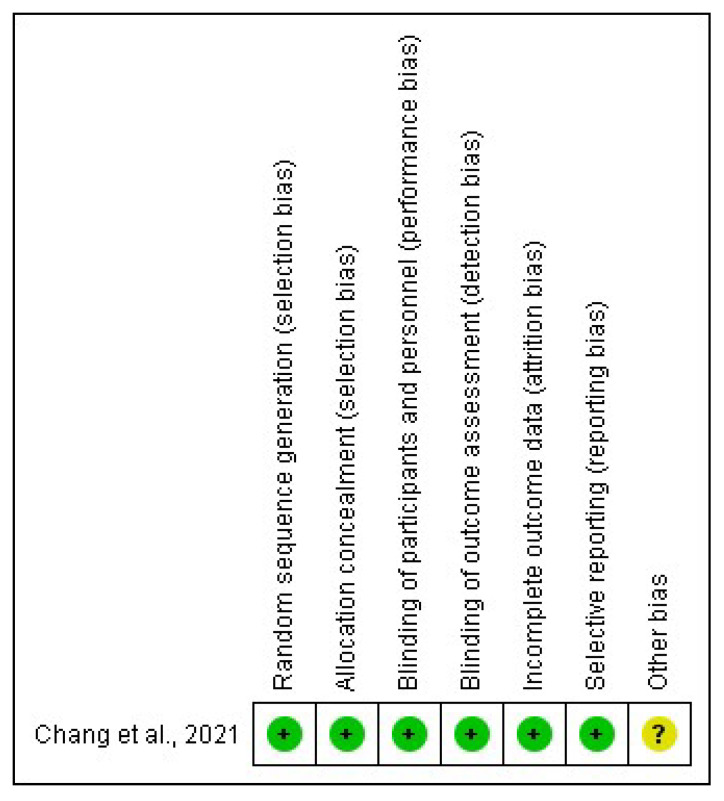
Risk of bias summary for the randomized controlled trial by Chang et al. (2021) [25] assessed using the Cochrane Risk of Bias 2.0 tool.

The study shows that the RCT was carefully planned, carried out, and had a minimal risk of bias in all important areas (see Figure 2 and Figure 3).

## 4. Discussion

This systematic review demonstrates that treating rhinosinusitis significantly improves objective parameters of sleep-disordered breathing (SDB) and sinonasal inflammation, although subjective symptom relief remains inconsistent. Across the included studies, interventions consistently improved polysomnographic indices such as the apnea-hypopnea index (AHI) and reduced mucosal disease burden on endoscopic examination. Importantly, objective and subjective sleep outcomes demonstrated different patterns of response. Improvements were more consistently observed in objective polysomnographic measures, whereas patient-reported measures of sleep quality, sinonasal symptoms, and quality of life were more variable across studies.

Several studies highlighted robust postoperative or post-treatment improvements. Uz et al. [14] demonstrated that functional endoscopic sinus surgery (FESS) for chronic rhinosinusitis with nasal polyps (CRSwNP) significantly lowered nasal resistance and improved both the apnea-hypopnea index and total apnea index. These objective improvements were accompanied by decreased Pittsburgh Sleep Quality Index (PSQI) scores, indicating better restorative sleep. Similarly, Värendh et al. [16] observed that FESS significantly improved the Sino-Nasal Outcome Test (SNOT) and Epworth Sleepiness Scale (ESS) scores, resulting in enhanced nasal patency and a reduced risk of OSA. Rotenberg et al. [19] confirmed that post-FESS improvements in sleep quality were independent of perceived nasal obstruction, emphasizing that sleep recovery may result from reduced mucosal inflammation rather than airflow mechanics alone. These findings were further supported by Alt et al. [26], who reported a 2.2-point improvement in PSQI scores and increased odds of achieving good sleep quality post-surgery (OR 5.94; *p* < 0.001). Together, these surgical studies establish that FESS and related interventions improve both objective and subjective sleep parameters, although the magnitude of benefit differs by baseline disease severity and comorbid OSA.

Evidence from large observational studies also shows the complex interaction between rhinosinusitis and SDB. Garvey et al. [17] found that patients with concurrent CRS and OSA exhibited higher rates of hypertension, diabetes, and asthma, as well as greater use of antibiotics and steroids, compared with those with either condition alone. This suggests that comorbid OSA amplifies CRS disease burden. Tajudeen et al. [22] corroborated these findings, showing that while patients with OSA initially reported poorer quality of life (QOL), both OSA and non-OSA CRS patients experienced comparable postoperative improvement following FESS. Importantly, disease severity and OSA status did not predict postoperative QOL outcomes after adjustment for confounding factors, implying that surgery benefits both groups equally. These data indicate a bidirectional relationship in which SDB worsens inflammatory sinonasal disease, yet its control improves overall symptom burden.

From an immunologic perspective, Kim et al. [15] provided mechanistic insights by showing that eosinophilic chronic rhinosinusitis with nasal polyps (ECRSwNP) is associated with elevated levels of IL-4, IL-6, IL-13, IL-17A, and CCL-24, reflecting activation of type 1, 2, and 3 immune pathways. These cytokine elevations were strongly correlated with moderate-to-severe OSA, whereas non-eosinophilic CRSwNP (NECRSwNP) patients showed no similar association. This reinforces the role of airway inflammation and immune heterogeneity in sleep impairment, suggesting that disease phenotype may determine the extent of sleep improvement following treatment. Complementary findings by Moffa et al. [18] demonstrated that biologic therapy with mepolizumab effectively reduced eosinophil counts, nasal obstruction, and rhinorrhea while improving the SNOT-22 and Nasal Polyp scores. However, olfactory recovery remained limited, consistent with persistent mucosal remodeling in severe CRSwNP.

Several studies further clarified the relationship between CRS and sleep disturbance independent of OSA severity. Jiang et al. [21] found that 64.7% of CRS patients had coexisting OSA, but its severity did not correlate with the extent of sinus inflammation, suggesting independent pathophysiological mechanisms driving sleep disturbance. A subsequent study by Jiang and Liang [23] confirmed that FESS improves sleep-related quality of life across most AHI groups, except in patients with severe OSAS. The only significant predictor of postoperative improvement was a lower preoperative AHI, suggesting that surgical intervention is most effective in mild-to-moderate disease. Similarly, Hui et al. [24] identified racial and phenotypic disparities, noting that African American patients and those with CRS without nasal polyps had nearly double the odds of developing OSA compared to White patients and CRSwNP subtypes. These findings suggest that demographic and inflammatory factors should be integrated into preoperative screening and counseling.

While the inflammation of the nose and sinuses and upper airway obstruction are known to be two mechanisms associated with rhinosinusitis and its association with sleep disordered breathing, recent studies indicate that obstructive sleep apnea is a multifactorial condition where anatomical, inflammatory, neurobiological, and genetic factors all interact. Thus, the benefits obtained from treating rhinosinusitis need to be considered in this broader context [29].

The influence of adjunctive therapies was also evaluated. Chang et al. [25] reported that postoperative oral prednisone provided no additional benefit in patients with CRSsNP undergoing ESS; rather, it was associated with worse psychological domain scores on SNOT-22. This highlights the potential risk–benefit imbalance of systemic corticosteroid uses in postoperative care. Similarly, Poachanukoon et al. [20] demonstrated that both acute and chronic rhinosinusitis responded well to antibiotic therapy alone, with notable symptom resolution and no surgical requirement. These findings underscore that medical management remains effective in milder or allergic subtypes of rhinosinusitis, while surgery should be reserved for refractory or anatomically obstructive disease.

Kim et al. [27] provided further evidence that isolated nasal surgery (septoplasty with turbinate reduction) may improve sleep-related symptoms such as snoring, fatigue, and apnea in patients with structural nasal obstruction, particularly when allergic rhinitis coexists. However, the overall surgical success rate was modest (14.3%), increasing to 50% among patients with moderate-to-severe obstruction, emphasizing the importance of careful patient selection. Finally, Laababsi et al. [28] reported that postoperative QOL improvement after FESS was most pronounced in patients with higher preoperative SNOT-22 scores (>20), while those with milder disease showed limited gains. Psychological and rhinologic symptom domains were the strongest predictors of meaningful clinical improvement, underscoring the necessity of individualized outcome expectations and preoperative evaluation.

### 4.1. Clinical Implications

The synthesized evidence base appears to support the use of functional endoscopic sinus surgery as the most effective approach for improving objective measures of SDB in patients with rhinosinusitis, especially when the phenotype includes nasal polyps or moderate to severe rhinosinusitis. Biologic therapy with mepolizumumab appears to hold promise for the subgroup of patients with severe eosinophilic CRSwNP, although the effect on polysomnographic parameters needs to be further explored. Nasal surgery alone appears to benefit selected patients with structural disease and allergic rhinitis. In contrast, the use of oral steroids appears to confer no benefit on SDB and may actually cause harm for the subgroup of patients with CRSsNP.

### 4.2. Strengths and Limitations

This SR has a number of strengths, including the evaluation of all aspects of the disease, including both medical and surgical approaches, and the use of validated tools such as the PSQI, ESS, and SNOT-22, among others. In addition, the use of objective outcomes such as the AHI and endoscopic scores also supports the positive impact of the treatment of sinonasal inflammation on the outcomes of sleep and airway function. Nevertheless, a number of limitations were also identified, including the high degree of clinical and methodological diversity of the studies, which makes it difficult to make comparisons between the studies. Most relied on measures that showed considerable inconsistency, and the overall methodological quality was moderate to serious, with no study achieving a low ROBINS-I risk rating. Acute and chronic rhinosinusitis, CRSwNP and CRSsNP phenotypes, pediatric and adult populations, and diverse interventions—including functional endoscopic sinus surgery, systemic corticosteroids, biologics, and antibiotics—were evaluated across studies. This variability limits direct comparability and precludes definitive pooled conclusions. As such, observed associations should be contextualized within specific disease phenotypes and treatment modalities rather than interpreted as uniform effects across all rhinosinusitis populations. Furthermore, because most published studies did not meet the predefined eligibility criteria, evidence regarding certain clinical interventions—particularly allergen desensitization immunotherapy and other targeted treatments for allergic multimorbidity—could not be included in this review. Consequently, the findings may not fully reflect the potential contribution of these therapies to improving sleep-related outcomes in patients with rhinosinusitis and associated allergic conditions. Hence, it is important to perform higher-quality randomized trials to clarify the effectiveness of integrated treatment approaches for rhinosinusitis-related sleep disturbances. Additionally, a funnel plot was not conducted due to the heterogeneity in outcomes, which would make interpretation of publication bias unreliable.

Although some studies have demonstrated positive outcomes in sleep-related parameters after the treatment of rhinosinusitis, these results should be interpreted with some caution. None of the studies included in this review demonstrated a low risk of bias. Many of the studies included in this review have been associated with confounding effects, selection bias, and subjective outcome measures. Therefore, although the results of this review indicate an association between reduced levels of sinonasal inflammation and sleep parameters, the strength of this association may still be limited. In addition, other limitations of this review include the fact that some of the studies have not consistently reported objective polysomnographic parameters. Furthermore, demographic factors, such as sex and comorbidity, have not been consistently described. In addition, the variation in obstructive sleep apnea-related parameters, which may include the measurement of AHI in the laboratory and at home, may have contributed to the variation in the results of this review. Finally, the broad eligibility criteria introduced substantial clinical and methodological heterogeneity, limiting direct comparisons between studies and precluding quantitative meta-analysis. In addition, a formal GRADE assessment was not performed because the review was based on a qualitative synthesis of highly heterogeneous studies. Therefore, the findings should be interpreted with caution.

## 5. Conclusions

In conclusion, this systematic review suggests that targeted treatment of rhinosinusitis, particularly endoscopic sinus surgery (ESS) and biologic therapy, may be associated with improvements in objective measures of sleep-disordered breathing, including the apnea-hypopnea index and endoscopic inflammatory scores. These findings highlight the importance of recognizing and managing sinonasal inflammation as a potentially modifiable contributor to sleep-disordered breathing. Future prospective studies and randomized controlled trials are needed to determine the long-term effects of rhinosinusitis treatment on sleep outcomes.

## Figures and Tables

**Table 1:** Characteristics and key findings of the 15 studies included in the systematic review evaluating rhinosinusitis treatment outcomes on sleep-disordered breathing.

Authors (Year)	Study Type	Intervention	Sample Size (*n*)	Outcome (Results)	Conclusion
Uz et al., 2017 [14]	Not mentioned	Surgical (FESS)	22	After surgery, nasal resistance dropped (*p* < 0.001). Postoperative PSQI ratings were significantly lower than preoperative ones (*p* < 0.001). The apnea-hypopnea index and total apnea index had preoperative mean values of 13.3 and 25.4, respectively. The total apnea and apnea-hypopnea index significantly decreased to 7.8 and 11.2, respectively, after surgery (*p* = 0.009 and 0.019, respectively).	For those with CRSwNP, functional endoscopic sinus surgery enhances sleep quality and patterns. Sleep-related respiratory disorders may be associated with CRSwNP.
Kim et al., 2021 [15]	Prospective observational study	Observational study (no intervention)	63	Patients with moderate-to-severe obstructive sleep apnea who had eosinophilic chronic rhinosinusitis with nasal polyps (ECRSwNP) showed higher levels of interleukin-6 and CXCL-1. IL-4, IL-13, CCL-11, CCL-24, and IL-17A were all raised in these people. On the other hand, transforming growth factor-beta expression was higher in individuals with moderate-to-severe OSA, but no significant differences in immune-related markers were seen in non-eosinophilic chronic rhinosinusitis with nasal polyps (NECRSwNP) in relation to OSA severity. Additionally, associations between raised inflammatory markers and serum indicators were discovered in ECRSwNP patients.	In ECRSwNP patients, OSA may enhance immunological profile heterogeneity (types 1, 2, and 3), but not in NECRSwNP patients.
Värendh et al., 2017 [16]	Prospective observational study	Functional endoscopic sinus surgery (FESS)	42	All sleep questionnaires revealed an effect on sleep patterns, and surgery unquestionably enhanced the quality of sleep. The median score on the Sino-Nasal Outcome Exam dropped from 51.5 to 26.5. The Epworth drowsiness scale score decreased from 7.5 to 6.0. In 13 patients assessed using the Berlin Questionnaire and Multivariable Apnea Prediction Index, surgery also decreased the likelihood of obstructive sleep apnea.	Following corrective surgery, patients with CRSwNP showed improvements in their nasal patency, sleep apnea risk, daytime drowsiness, and poor sleep quality.
Garvey et al., 2024 [17]	Retrospective cohort study using large electronic health record database (TriNetX)	Medical (CPAP therapy) or surgical (UPPP, tonsillectomy, hypoglossal nerve stimulation) interventions for OSA	1,818,879 CRS- only patients	The incidences of hypertension, diabetes, obesity, and asthma were found to be higher in CRS-OSA patients than in CRS or OSA patients (*p* < 0.0001) in a study that included 1,818,879 patients with chronic rhinosinusitis (CRS), 481,144 patients with obstructive sleep apnea (OSA), and 93,153 patients with both conditions (CRS-OSA). They also had greater rates of endoscopic sinus surgery (ESS) (OR: 1.91), antibiotic use (OR: 1.90), and oral steroid treatment (OR: 2.23) than individuals with CRS alone. Patients with CRS-OSA who did not employ continuous positive airway pressure were substantially more likely to take antibiotics (OR: 3.24) and steroids (OR: 2.28) than those who did. Additionally, CRS-OSA patients undergoing sleep-related surgery required fewer courses of antibiotics (OR: 1.93).	Comorbidities associated with both disorders were more common in CRS-OSA patients than in those with CRS or OSA alone. OSA was associated with an increased risk of ESS, antibiotic use, and steroid use in people with CRS. There appears to be a link between OSA therapy and CRS results, but more research is required.
Moffa et al., 2025 [18]	Prospective observational study	Treated with mepolizumab for 12 months	20 (all sample)	At the end of the enrollment phase, twenty people with severe CRSwNP were enrolled. Olfactory function did not significantly improve (*p* < 0.05), while nasal obstruction and rhinorrhea (*p* < 0.05), quality of life (SNOT-22; *p* < 0.05), and Nasal Polyp Score all showed notable improvements. Eosinophil counts also significantly decreased (*p* < 0.05).	Mepolizumab enhances quality of life and symptom control in patients with severe CRSwNP and has an acceptable safety profile.
Rotenberg et al., 2015 [19]	Prospective cohort study	FESS	53 (all sample)	Fifty-three patients satisfied the requirements for inclusion and exclusion. There was a statistically and clinically significant improvement in sleep outcomes (PSQI before FESS = 10.9 ± 2.8, PSQI after FESS = 5.3 ± 2.2, *p* < 0.01; EpSS before FESS = 14.7 ± 3.1, EpSS after FESS = 9.1 ± 1.1, *p* < 0.01). Additionally, CRS-specific results were enhanced. Scores for nasal blockage did not alter much.	The individuals in our study had better sleep results because of FESS. Correcting the nasal blockage had no bearing on this. Sleep is positively impacted by sinus surgery for CRSsNP; this new information can be utilized in patient counseling and as justification to third-party payers.
Poachanukoon et al., 2012 [20]	Observational, cross-sectional comparative study	Medical treatment with antibiotics (primarily amoxicillin- clavulanic acid and cefditoren pivoxil)	154 (103 ARS, 51 CRS)	Cough and rhinorrhea were the primary complaints in both acute and chronic cases of allergic rhinitis, which was common in individuals with chronic rhinosinusitis (CRS). There were notable variations in the CRS group’s symptoms, such as increased periorbital discomfort and sleep apnea. 65% of individuals with acute rhinosinusitis (ARS) and 58.8% of patients with CRS had abnormal X-ray results; the CRS group had adenoid hypertrophy. After receiving antibiotics for an average of 14.6 days for ARS and 22.35 days for CRS, both groups exhibited recovery, and none of them needed sinus surgery.	Rhinorrhea and cough were the most prevalent signs of RS. Chronic rhinosinusitis was more common in patients with allergic rhinitis. Patients with CRS and ARS received effective medical care.
Jiang et al., 2016 [21]	Prospective study (data collected from CRS patients undergoing functional endoscopic sinus surgery)	Functional endoscopic sinus surgery (FESS) (performed on CRS patients unresponsive to medical therapy).	139 (all diagnosed with chronic rhinosinusitis [CRS]	Of them, 38.1% reported feeling sleepy during the day, and nasal obstruction was linked to this sleep issue. 64.7% of the patients had a diagnosis of obstructive sleep apnea syndrome (OSAS), although there was no connection between the severity of rhinosinusitis and OSAS. In patients with CRS, nasal polyps did not exacerbate sleep issues.	According to this study, OSAS was highly prevalent in CRS patients, and there was no correlation between the severity of rhinosinusitis and worse OSAS.
Tajudeen et al., 2017 [22]	Multi-institution, retrospective cohort study	Functional endoscopic sinus surgery (FESS) for medically recalcitrant chronic rhinosinusitis (CRS).	480 (all had chronic rhinosinusitis and underwent FESS)	Following surgery, patients with and without OSA reported a significant improvement in quality of life (*p* < 0.0001) compared to baseline. The 22-Item Sino-Nasal Outcome Test scores at each time point in the unadjusted model were substantially poorer for the participants with OSA (2.4 points higher per time point, *p* = 0.006). The adjusted model revealed no difference in QOL outcome based on OSA status after adjusting for variables (*p* = 0.114). The adjusted model revealed no variation in the QOL result when OSA disease severity was stratified.	After FESS, patients with CRS and concomitant OSA had lower QOL outcomes; however, there was no difference in QOL outcomes when patient variables were taken into account. QOL improvement with FESS did not appear to be predicted by the severity of OSA disease.
Jiang et al., 2019 [23]	Prospective observational study	Functional endoscopic sinus surgery (FESS)	96 (all participants had CRS)	After FESS, all AHI groups—aside from the severe OSAS group—saw a decline in their sleep domain scores on the SNOT-20 and ESS. After FESS, nine patients (13.2%) had an AHI drop of less than five.	Our findings demonstrated that FESS enhanced sleep quality in CRS patients, with the exception of those with severe OSAS, and that the only meaningful predictor of post-FESS OSAS outcome was a lower AHI prior to surgery.
Hui et al., 2017 [24]	Retrospective cohort study	No intervention—observational only	916 (entire sample had CRS)	A multivariable regression model was used to show that, with an adjusted odds ratio of 1.98, African American patients are more likely than white patients to develop obstructive sleep apnea (OSA). With an odds ratio of 1.63, people with chronic rhinosinusitis (CRS) who do not have nasal polyps are also more likely to develop OSA.	Compared to white patients, African American patients with CRS were more likely to develop OSA, hence OSA screening is necessary for this patient population.
Chang et al., 2021 [25]	Prospective, double-blind, placebo-controlled, randomized clinical trial	Postoperative oral corticosteroids (prednisone taper) after endoscopic sinus surgery	72 (all participants had CRSsNP and underwent ESS)	There were no clinically significant variations in the Lund–Kennedy or SNOT-22 total scores when comparing longitudinal differences between treatment groups. Additionally, rhinologic, extranasal rhinologic, ear/facial, and sleep subdomain assessments of the SNOT-22 revealed no significant changes. Interestingly, compared to the placebo group, the prednisone group scored lower on psychological dysfunction. The two therapy groups saw similar side effects.	According to SNOT-22 total scores, rhinologic subscores, and Lund–Kennedy endoscopy scores over a six-month period, patients with chronic rhinosinusitis (CRS) without polyps did not benefit more from oral prednisone after endoscopic sinus surgery (ESS) than from a placebo in a randomized clinical trial. Prednisone users significantly performed worse on the SNOT-22 psychologic subcategory. These results suggest that oral corticosteroids may have more dangers than benefits, thus their use after ESS for CRS without polyps should be carefully considered.
Alt et al., 2014 [26]	Prospective observational study	Endoscopic sinus surgery	342	Surgery significantly improved all seven subdomain scores as well as the overall mean global PSQI scores by 2.2 points. Patients undergoing sinus surgery had a significantly higher chance of having good sleep quality (PSQI ≤ 5) (odds ratio 5.94; 95% CI, 3.06 to 11.53; *p* < 0.001). Acetylsalicylic acid intolerance (β −1.94; 95% CI, −3.77 to −0.11; *p* = 0.038), history of sinus surgery (β 1.10; 95% CI, 0.03 to 2.16; *p* = 0.044), and frontal sinusotomy (β −1.03; 95% CI, −2.26 to 0.20; p = 0.099) were found to be significant factors linked to an improvement in PSQI sleep scores.	Following ESS, patients with CRS showed improvements in their disease severity, poor disease-specific quality of life, and decreased sleep quality.
Kim et al., 2021 [27]	Prospective, nonrandomized study	Septoplasty with volume reduction in the inferior turbinate on both sides, performed using a microdebrider.	-	Patients’ symptoms, including snoring, sleep apnea, morning headaches, fatigue, and daytime sleepiness, significantly improved six months after surgery. The findings of the polysomnography showed improvements in the respiratory disturbance index, apnea-hypopnea index, and duration of oxygen saturation below 90%. Nasal surgery had an overall success rate of 14.3%, but it rose to 50% in patients with allergic rhinitis and was significantly greater in those with moderate to severe nasal obstruction than in those with mild obstruction.	Patients with significant nasal blockage and allergic rhinitis are more likely to have a better surgical outcome after solitary nasal surgery.
Laababsi et al., 2019 [28]	Prospective cohort	FESS	72	Preoperative and postoperative SNOT-22 scores improved more significantly in the U CRSsNP group (37.13 ± 9.307 compared 14.11 ± 8.531) and the B CRSsNP group (41.76 ± 6.949 versus 18.57 ± 8.495). In the U CRSsNP group, 88% of patients with preoperative SNOT-22 scores greater than 20 points achieved MCID. In the other group, 66% of patients with a preoperative SNOT-22 score of more than 40 points had MCID. Preoperative factors that affect QOL outcomes were identified using a multivariate logistic regression model.	According to the study’s findings, there was no likelihood of an MCID improvement following FESS for patients with U CRSsNP who had preoperative SNOT-22 scores between 10 and 19 and patients with B CRSsNP who had preoperative SNOT-22 scores between 10 and 19 or 20–29. In contrast to the unilateral nature of CRS, preoperative rhinologic symptoms and preoperative psychological dysfunction areas of SNOT-22 are useful instruments to predict improvement with FESS.

**Table 2:** Risk of bias assessment for non-randomized studies using the ROBINS-I (Risk Of Bias In Non-randomized Studies of Interventions) tool.

Authors	D1: Confounding	D2: Selection	D3: Intervention Classification	D4: Deviations	D5: Missing Data	D6: Outcome Measurement	D7: Reported Result	Overall Risk
Uz et al., 2017 [14]	Serious	Moderate	Low	Low	Low	Moderate	Low	Serious
Värendh et al., 2017 [16]	Moderate	Low	Low	Low	Moderate	Moderate	Low	Moderate
Garvey et al., 2024 [17]	Moderate	Moderate	Low	Low	Moderate	Moderate	Low	Moderate
Moffa et al., 2025 [18]	Serious	Low	Low	Moderate	Moderate	Moderate	Low	Serious
Rotenberg et al., 2015 [19]	Serious	Moderate	Low	Low	Low	Moderate	Low	Serious
Tajudeen et al., 2017 [22]	Moderate	Low	Low	Moderate	Moderate	Moderate	Low	Moderate
Jiang et al., 2019 [23]	Moderate	Moderate	Low	Low	Low	Low	Low	Moderate
Alt et al., 2014 [26]	Serious	Low	Low	Moderate	Moderate	Moderate	Low	Serious
Kim et al., 2021 [27]	Serious	Low	Low	Moderate	Low	Low	Low	Serious

**Table 3:** Newcastle–Ottawa Scale (NOS) risk of bias assessment for observational cohort and cross-sectional studies (*n* = 5).

Authors	Q1	Q2	Q3	Q4	Q5	Q6	Q7	Q8	Risk of Bias Scores (0–2: High, 3–5: Moderate, 6–8: Low)	Quality Score
Hui et al., 2017 [24]	1	1	1	0	2	1	0	0	6/9	Moderate risk of bias
Kim et al., 2021 [15]	1	1	1	0	**0**	0	1	1	5/9	Moderate Risk
Poachanukoon et al., 2012 [20]	1	1	1	0	**0**	0	0	0	3/9	High Risk
Jiang et al., 2016 [21]	1	1	1	1	1	0	1	1	7/9	Low Risk
Laababsi et al., 2019 [28]	1	1	1	1	0	0	1	1	6/9	Moderate Risk

## Data Availability

All data generated or analyzed during this study are included in this published article. Additional datasets or Supplementary Materials related to this systematic review are available from the corresponding author upon reasonable request.

## Supplementary Materials

The following supporting information can be downloaded at: https://www.mdpi.com/article/10.3390/medicina62071392/s1, PRISMA 2020 Checklist [10].
